# Effects of varying blood flow rate during peripheral veno-arterial extracorporeal membrane oxygen (V-A ECMO) on left ventricular function measured by two-dimensional strain

**DOI:** 10.3389/fcvm.2023.1147783

**Published:** 2023-04-12

**Authors:** Pauline Yeung Ng, Tammy Sin Kwan Ma, April Ip, Shu Fang, Andy Chak Cheung Li, Alfred Sai Kuen Wong, Chun Wai Ngai, Wai Ming Chan, Wai Ching Sin

**Affiliations:** ^1^ Critical Care Medicine Unit, School of Clinical Medicine, The University of Hong Kong, Hong Kong, Hong Kong SAR, China; ^2^ Department of Adult Intensive Care, Queen Mary Hospital, Hong Kong, Hong Kong SAR, China; ^3^ Department of Medicine, The University of Hong Kong, Hong Kong, Hong Kong SAR, China

**Keywords:** extracorporeal membrane oxygenation, target blood flow, transthoracic echocardiography, cardiogenic shock, myocardial strain, speckle tracking echocardiography

## Abstract

**Background:**

We evaluated the effects of varying blood flow rate during peripheral veno-arterial extracorporeal membrane oxygen (V-A ECMO) on left ventricular function measured by two-dimensional strain.

**Methods:**

Adult patients who were supported by peripheral V-A ECMO were recruited. Serial hemodynamic and cardiac performance parameters were measured by transthoracic echocardiogram within the first 48 h after implementation of V-A ECMO. Measurements at 100%, 120%, and 50% of target blood flow (TBF) were compared.

**Results:**

A total of 54 patients were included and the main indications for V-A ECMO were myocardial infarction [32 (59.3%)] and myocarditis [6 (11.1%)]. With extracorporeal blood flow at 50% compared with 100% TBF, the mean arterial pressure was lower [66 ± 19 vs. 75 ± 18 mmHg, *p* < 0.001], stroke volume was greater [23 (12–34) vs. 15 (8–26) ml, *p* < 0.001], and cardiac index was higher [1.2 (0.7–1.7) vs. 0.8 (0.5–1.3) L/min/m^2^, *p* < 0.001]. Left ventricular contractile function measured by global longitudinal strain improved at 50% compared with 100% TBF [−2.8 (−7.6- −0.1) vs. −1.2 (−5.2–0) %, *p* < 0.001]. Similarly, left ventricular ejection fraction increased [24.4 (15.8–35.5) vs. 16.7 (10.0–28.5) %, *p* < 0.001] and left ventricular outflow tract velocity time integral increased [7.7 (3.8–11.4) vs. 4.8 (2.5–8.5) cm, *p* < 0.001]. Adding echocardiographic parameters of left ventricular systolic function to the Survival After Veno-arterial ECMO (SAVE) score had better discriminatory value in predicting eventual hospital mortality (AUROC 0.69, 95% CI 0.55–0.84, *p* = 0.008) and successful weaning from V-A ECMO (AUROC 0.68, 95% CI 0.53–0.83, *p* = 0.017).

**Conclusion:**

In the initial period of V-A ECMO support, measures of left ventricular function including left ventricular ejection fraction and global longitudinal strain were inversely related to ECMO blood flow rate. Understanding the heart-ECMO interaction is vital to interpretation of echocardiographic measures of the left ventricle while on ECMO.

## 1. Introduction

The use of veno-arterial extracorporeal membrane oxygenation (V-A ECMO) as a form of temporary mechanical circulatory support has increased exponentially over the past decade (1). Due to its relative ease of setting up, peripheral V-A ECMO has become a readily available option to facilitate resuscitation at the bedside. A randomized controlled trial showed that early ECMO-facilitated resuscitation for patients with out-of-hospital cardiac arrest significantly improved survival to hospital discharge (2).

In the peripheral configuration of V-A ECMO, oxygenated blood from the ECMO circuit returns to the arterial system in a “retrograde” manner from cannulas sited in peripheral vasculature, most commonly the femoral artery. This returning blood flow may increase the left ventricular (LV) afterload, leading to increase in LV wall stress and myocardial oxygen demand, a condition that may be deleterious to the recovery of an acutely-injured heart (3, 4). Although this phenomenon has been postulated, the magnitude of changes in LV performance parameters in response to different levels of ECMO blood flow during the immediate period after initiation have not been well-delineated in prospective cohorts, and the lack of such fundamental physiological data regarding the heart-ECMO interaction may be partially accountable for the difficulty in establishing recommendations for target flow rates. Two-dimensional strain measured by echocardiography has become an increasing utilized tool in the intensive care unit for assessment of LV function (5), and holds advantage over traditional tools like the pulmonary arterial catheter in being non-invasive and repeatable.

In this prospective observational study, we utilized detailed echocardiography and strain analyses to examine LV function in response to different levels of ECMO blood flow during acute cardiogenic shock. We hypothesized that the association between LV systolic function and ECMO blood flow rate during the initial period of peripheral V-A ECMO support can be measured by two-dimensional strain.

## 2. Materials and methods

### 2.1. Study population

This was a single-center prospective observational study including all adult patients (≥18 years old) with acute cardiogenic shock who were admitted between April 2019 and December 2021, and were treated with peripheral V-A ECMO in a tertiary referral center in Hong Kong.

Patients were excluded if they met one of the following criteria: (1) clinically unstable hemodynamics including unstable or poor ECMO blood flow which precludes ECMO flow adjustment; (2) presence of pathological intracardiac shunt, for example, ventricular septal defect; (3) presence of iatrogenic shunt, for example, left ventricular vent; (4) echocardiographic image quality unsatisfactory for data processing; or (5) absence of patient or surrogate consent. Patients who had concurrent use of intra-aortic balloon pump (IABP) were not excluded. This study was approved by the Institutional Review Board of the University of Hong Kong/Hospital Authority Hong Kong West Cluster (HKU/HA HKW IRB) (IRB Reference Number: UW 17–449). Informed consent was obtained from all participants or their surrogates if the fitness to consent was impaired.

### 2.2. Material, equipment and procedures

Transthoracic echocardiogram (TTE) examinations were performed as soon as practicable within the first 48 h after initiation of V-A ECMO support by one of the 3 physicians trained in detailed cardiac echocardiography, using a commercially available system (General Electric Healthcare Vivid q cardiovascular ultrasound system). Two-dimensional sequences with three beats were obtained using a 3.5 MHz ultrasound transducer probe at a frame rate of 50 frames/s and stored digitally in Digital Imaging and Communications in Medicine (DICOM) format. Standard echocardiographic measurements were obtained according to current recommendations (6), and repeated with ECMO flow settings at 100%, 120%, and 50% target blood flow (TBF) (defined as 50–80 ml/kg ideal body weight/min) (7, 8). At each ECMO blood flow setting, echocardiographic measurements were taken after 5 min of flow adjustment to allow equilibration. Measurements were abandoned and further flow adjustment not attempted if the patient developed clinically significant acute deterioration in hemodynamics. To minimize effects of pharmacologically-induced alterations in systemic vascular resistance, the titration of vasopressor administration during ECMO flow adjustment was not recommended.

LV systolic function assessment included traditional parameters of left ventricular ejection fraction (LVEF), fractional shortening (FS), left ventricular outflow tract (LVOT) velocity-time integral (VTI), left ventricular index of myocardial performance (LIMP), and peak systolic tissue velocity (s') at the mitral annulus measured by pulsed-wave Doppler. LVEF was measured using both the linear (Teichholz formula) and biplane Simpson methods. Two-dimensional strain for each of the LV segments were measured by speckle tracking echocardiography in longitudinal 3-chamber, 4-chamber and 2-chamber planes. The LV global longitudinal strain (GLS) was obtained by averaging segmental strain values. All echocardiographic measurements were analyzed offline by a single investigator blinded to clinical data and subsequent analyses. Detailed definitions of these measurements are shown in Supplementary Table S1.

Hemodynamic data were measured by continuous invasive measurement of arterial pressure and by parameters obtained during transthoracic echocardiography according to current recommendations (9). Patient management after initiation of V-A ECMO was according to standard practice. The readiness to wean from ECMO was determined by decremental flow studies.

### 2.3. Outcomes

The primary outcome was the LV systolic function measured by two-dimensional GLS. Secondary outcomes were other measures of LV systolic function, including LVEF using modified Simpson's rule, LVOT VTI, LIMP, and s' at the lateral and septal mitral annular level.

### 2.4. Statistical analysis

#### 2.4.1. Primary analysis

Data were expressed either as mean ± standard deviation or median with interquartile ranges for continuous variables, and frequencies with percentages for categorical variables. Comparisons between LV parameters at different ECMO flow rates were made using paired t-tests or Wilcoxon signed-rank tests.

#### 2.4.2. Secondary analysis

We examined whether data gathered early after ECMO implantation is useful to predict eventual clinical outcomes. The performance of 2 different models were assessed—Model 1 was the Survival After Veno-arterial ECMO (SAVE) score (10); Model 2 was the SAVE score together with echocardiographic parameters of LV systolic function obtained at 100% TBF, including LVEF measured by the biplane method, LVOT VTI, average s' at the mitral annulus, and LV GLS. The utility of these models to predict eventual hospital mortality and successful weaning from V-A ECMO were examined. Model calibration and discrimination were examined by the Hosmer-Lemeshow test and area under the receiver operating characteristic (AUROC) curve.

#### 2.4.3. Sensitivity analysis

It is possible that the concurrent use of IABP affects echocardiographic parameters of LV function, therefore, a subgroup analysis excluding patients on IABP during ECMO was performed.

Data management and statistical analyses were performed in Stata, version 13 (StataCorp LP). Bonferroni correction was used to adjust for multiple comparisons, and a two-tailed *P* value of less than 0.025 was considered statistically significant.

## 3. Results

### 3.1. Study population

**Figure 1:**
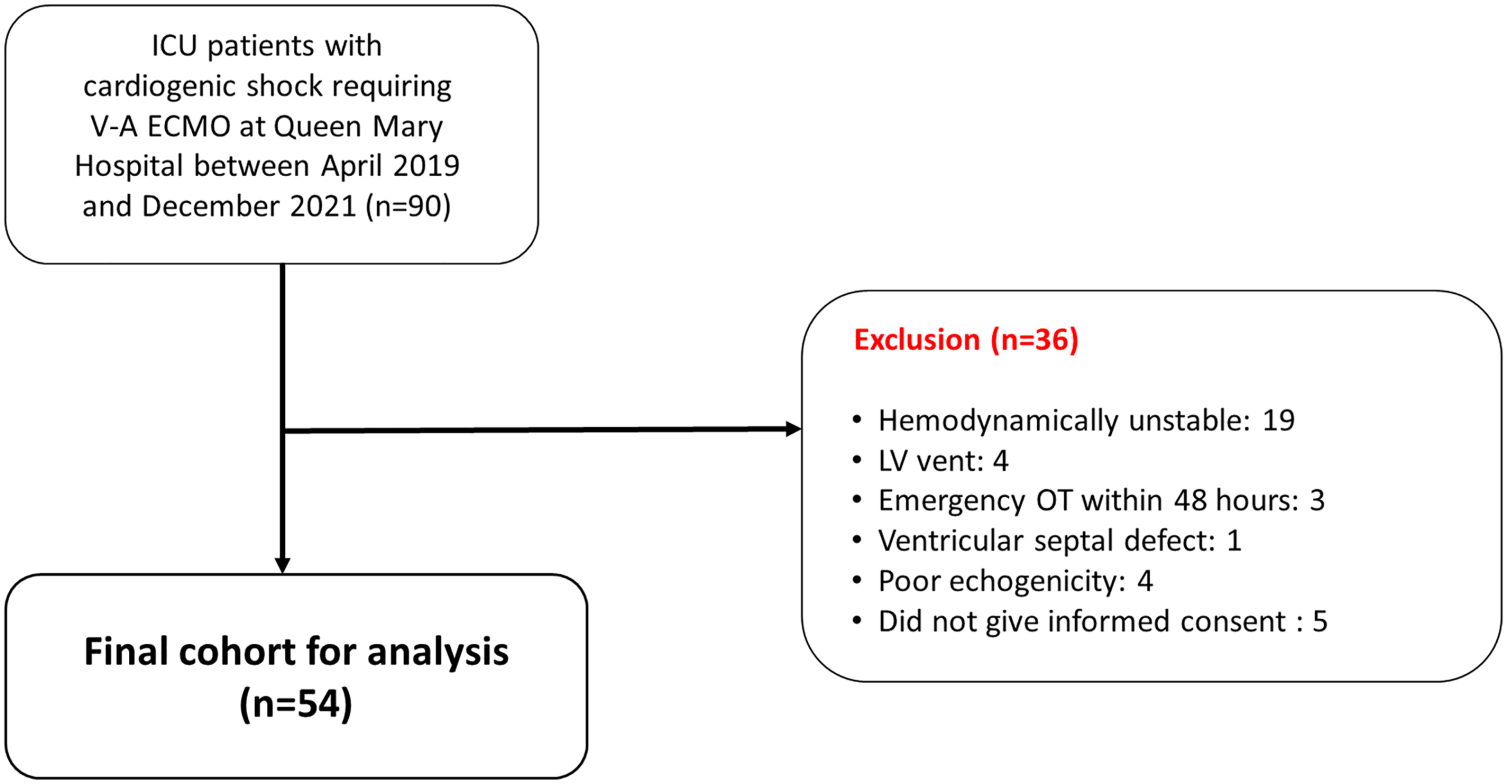
Flow diagram of the study. A total of 90 adult patients were admitted to the intensive care unit with a diagnosis of cardiogenic shock requiring V-A ECMO support between 1st April 2019 and 31st December 2021. After excluding 36 patients, the final patient cohort consisted of 54 patients. ICU, intensive care unit; LV, left ventricular; OT, operation; V-A ECMO, veno-arterial extracorporeal membrane oxygenation.

From 1st April 2019 to 31st December 2021, 90 adult patients who were admitted to the intensive care unit (ICU) with a diagnosis of cardiogenic shock requiring V-A ECMO support were identified (Figure 1). After excluding 19 patients with unstable hemodynamics and could not tolerate ECMO flow adjustment, 4 patients who had LV venting, 3 patients requiring urgent cardiotomy, 1 patient with ventricular septal defect, 4 patients who had unsatisfactory echocardiographic image quality, and 5 patients who did not give informed consent, the final patient cohort consisted of 54 patients.

The median age of the study population was 59 (50–65) years and there were 40 (74.1%) males. The most common indications of V-A ECMO were acute coronary syndrome (32, 59.3%) and myocarditis (6, 11.1%). Baseline characteristics are summarized in Table 1. A total of 37 (68.5%) patients received V-A ECMO during extracorporeal cardiopulmonary resuscitation (ECPR).

**Table 1:** Baseline characteristics of the patient population.

	*n* = 54
**Demographics**	
Age	59 (50–65)
Sex—male	40 (74.1%)
Mean body mass index, kg/m^2^	25.7 ± 4.7
Mean body surface area, m^2^	1.8 ± 0.2
Hospital stay before ECMO initiation, hours	0.7 (0–18.6)
**Co-morbidities**	
Active smoker	17 (31.5%)
Hypertension	18 (33.3%)
Diabetes mellitus	13 (24.1%)
Ischemic heart disease	9 (16.7%)
Valvular heart disease	1 (1.9%)
Cardiomyopathy	3 (5.6%)
**Indications for V-A ECMO**	
Acute coronary syndrome	32 (59.3%)
Myocarditis	6 (11.1%)
Malignant arrhythmia	5 (9.3%)
Others^a^	11 (20.4%)
**Clinical Scores**	
APACHE IV score	122.4 ± 28.5
APACHE II score	31.9 ± 7.9
SAVE score	−5.8 ± 5.5
Vasoactive Inotropic Score^b^	24.8 (6.6–48.7)
**Hemodynamic parameters** ^c^	
Mean arterial pressure, mmHg	55 (45–76)
Heart rate, beats per minute	115 (101–126)
**Biochemistry** **^c^**	
Creatinine, umol/L	204.5 (136.0–294.0)
Bilirubin, umol/L	19.8 (11.0–30.0)
Creatine Kinase, U/L	806.0 (224.0–3,124.0)
Troponin T, ng/L	7,680 (1,470–23,634)
Lactate, mmol/L	11.6 ± 5.9
PCI after ECMO established	23 (42.6%)

APACHE II score, acute physiology and chronic health evaluation II score; APACHE IV score, acute physiology and chronic health evaluation IV score; PCI, percutaneous coronary intervention; SAVE, survival after Veno-Arterial ECMO score; V-A ECMO, veno-arterial extracorporeal membrane oxygenation.

All data are presented as frequency with percentages or mean ± standard deviation, or median with interquartile range (IQR) unless specified.

^a^ Other indications for V-A ECMO included endocrine-related heart failure and decompensated heart failure.

^b^ The Vasoactive Inotropic Score was calculated as: Dopamine dose (μg/kg/min) + Dobutamine dose (μg/kg/min) + 100 × Adrenaline dose (μg/kg/min) + 10 × Milrinone dose (μg/kg/min) + 10,000 × Vasopressin dose (unit/kg/min) + 100 × Noradrenaline dose (μg/kg/min).

^c^ Worst values on day 1 of ECMO support.

On day 1 of ECMO support, the median of the lowest mean arterial pressure (MAP) was 55 (45–76) mmHg and the highest heart rate were 115 (101–126) bpm. The vasoactive inotropic score was 24.8 (6.6–48.7), and the mean of the highest lactate level was 11.6 ± 5.9 mmol/L. The mean APACHE IV score was 122.4 ± 28.5. Detailed clinical parameters are summarized in Supplementary Table S2.

### 3.2. Clinical outcomes

The median duration on V-A ECMO was 120.3 (70.3–188.0) hours. There were 25 (46.3%) patients who were able to be weaned off ECMO. A total of 25 (46.3%) patients survived the ICU stay and 23 (42.6%) patients survived the hospital stay. There were 2 (3.7%) patients who had IABP inserted prior to V-A ECMO, and 5 (9.3%) patients required IABP insertion after ECMO was initiated. The clinical outcomes stratified by SAVE score are shown in Supplementary Table S3.

### 3.3. Hemodynamic data

There were significant differences in hemodynamic parameters with ECMO blood flows at 50% compared with 100% and 120% TBF. The MAP was significantly lower at 50% compared with 100% TBF, and at 50% compared with 120% TBF (66 ± 19 vs. 75 ± 18 mmHg, *p* < 0.001; 66 ± 19 vs. 77 ± 17 mmHg, *p* < 0.001; respectively). Having ECMO blood flow at 50% TBF when compared with 100% TBF and 120% TBF was associated with higher stroke volume (SV) [23 (12–34) vs. 15 (8–26) ml, *p* < 0.001; 23 (12–34) vs. 12 (6–21) ml, *p* < 0.001], and higher cardiac index (CI) [1.2 (0.7–1.7) vs. 0.8 (0.5–1.3) L/min/m^2^, *p* < 0.001; 1.2 (0.7–1.7) vs. 0.6 (0.3–1.3) L/min/m^2^, *p* < 0.001], respectively.

### 3.4. Two-Dimensional myocardial strain measurements

TTE were performed on a mean 1.0 ± 0.7 days after initiation of ECMO. The myocardial systolic function measured by LV GLS was significantly better at 50% TBF compared with 100% TBF and 120% TBF [−2.8 (−7.6- −0.1) vs. −1.2 (−5.2–0) %, *p* < 0.001; −2.8 (−7.6–−0.1) vs. 0 (−3.8–0) %, *p* < 0.001], respectively. Similarly, there were significant differences in segmental strain values obtained at the LV longitudinal 3-chamber, 2-chamber, and 4-chamber views when ECMO blood flow was titrated.

### 3.5. Left ventricular systolic function measurements

**Figure 2:**
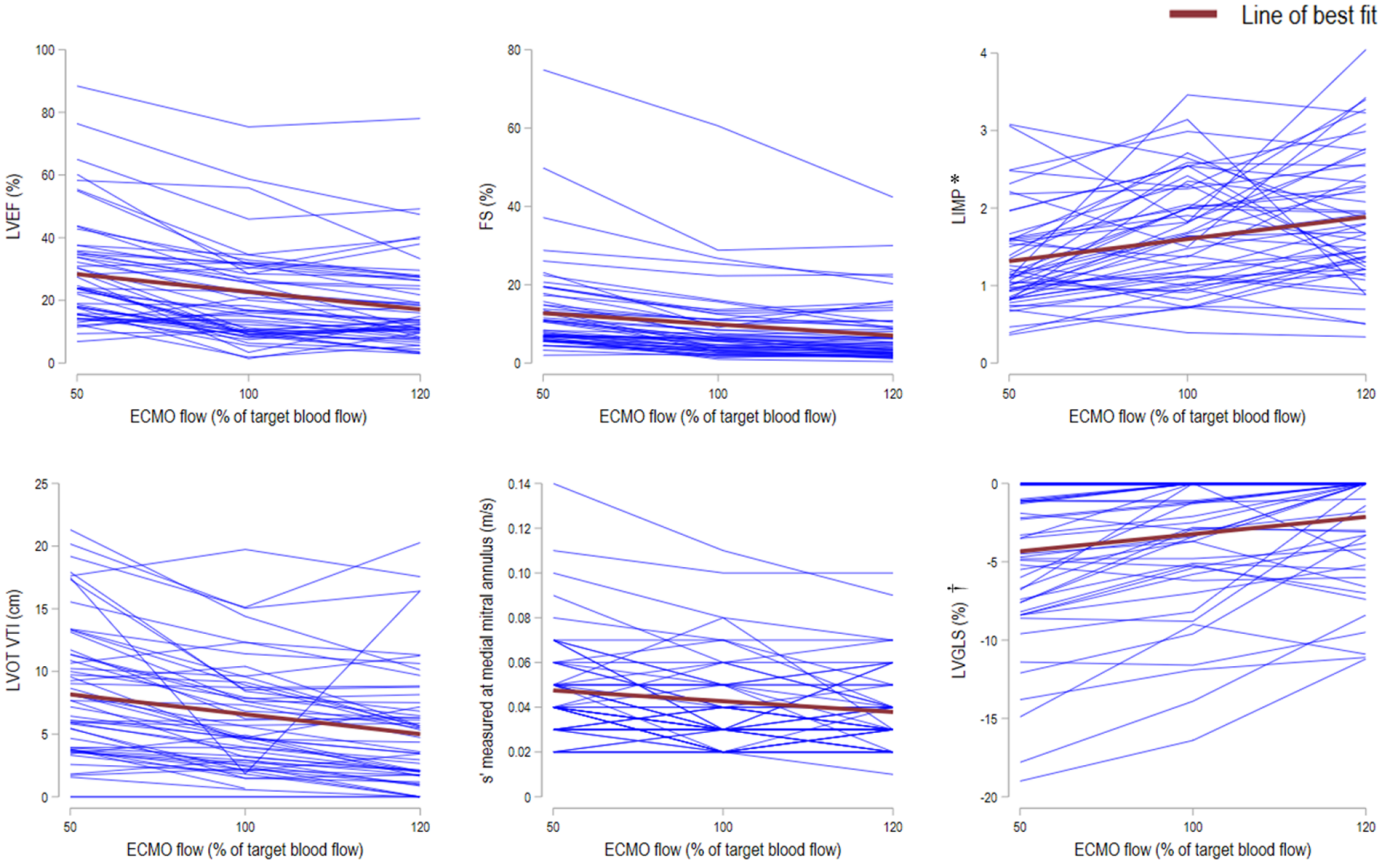
Spaghetti plots for various measures of left ventricular function against ECMO target blood flow. The plots of LVEF (left ventricular ejection fraction; top left), FS (fractional shortening; top middle), LIMP (left ventricular index of myocardial performance; top right), LVOT VTI (left ventricular outflow tract velocity time integral; bottom left), s’ (peak systolic tissue velocities measured at medial mitral annulus; bottom middle), and LV GLS (left ventricular global longitudinal strain; bottom right) against ECMO target blood flow of 50%, 100%, and 120% (*n* = 54). Each blue line represents an individual participant. The line of best fit for each plot is shown in red. * LIMP: higher values indicate more impaired systolic function. † GLS (%): the “negative” sign indicates myocardial shortening. Higher negative % values indicate more pronounced longitudinal shortening, whereas values closer to zero indicate less shortening (i.e., more impaired systolic function).

The LV systolic function was significantly better across all echocardiographic measurements at 50% TBF compared with 100% TBF, and at 50% TBF compared with 120% TBF. These included the LVEF measured by the biplane method [24.4 (15.8–35.5) vs. 16.7 (10.0–28.5)%, *p* < 0.001; 24.4 (15.8–35.5) vs. 13.4 (9.6–26.3)%, *p* < 0.001], FS [9.5 (6.7–15.7) vs. 6.0 (3.3–10.9)%, *p* < 0.001; 9.5 (6.7–15.7) vs. 4.8 (2.5–9.3)%, *p* < 0.001], LIMP [1.1 (0.8–1.6) vs. 1.6 (1.1–2.3), *p* < 0.001; 1.1 (0.8–1.6) vs. 1.7 (1.2–2.4), *p* < 0.001], LVOT VTI [7.7 (3.8–11.4) vs. 4.8 (2.5–8.5) cm, *p* < 0.001; 7.7 (3.8–11.4) vs. 4.4 (1.8–7.2) cm, *p* < 0.001], and s' measured at the medial [0.04 (0.03–0.06) vs. 0.03 (0.03–0.05) m/s, *p* = 0.001; 0.04 (0.03–0.06) vs. 0.03 (0.03–0.05) m/s, *p* < 0.001] and lateral mitral annulus [0.05 (0.03–0.06) vs. 0.04 (0.03–0.06) m/s, *p* = 0.011; 0.05 (0.03–0.06) vs. 0.04 (0.03–0.05) m/s, *p* < 0.001]. Detailed echocardiographic data at different levels of ECMO blood flows are shown in Table 2, and stratified by hospital mortality in Supplementary Table S4. Spaghetti plots for various measures of LV function against ECMO TBF are shown in Figure 2.

**Table 2:** Comparison of Echo findings at different levels of ECMO target blood flow.

ECMO Target Blood Flow (*n* = 54)					
	100%	120%	50%	*P* value for 100% and 50%	*P* value for 120% and 50%
**Left ventricle size**					
LVIDd, cm	4.2 (3.6–4.9)	4.1 (3.3–4.9)	4.0 (3.3–4.9)	*0*.*006*	0.15
LVIDs, cm	4.0 (3.2–4.8)	3.7 (3.1–4.7)	3.6 (2.8–4.3)	*<0*.*001*	*<0*.*001*
LVEDV, ml	74.3 (55.5–116.6)	78.0 (45.3–109.4)	80.2 (41.0–119.2)	0.58	0.69
LVESV, ml	58.3 (30.4–92.7)	58.8 (32.8–100.7)	51.6 (26.3–93.1)	*0*.*002*	*0*.*002*
**Left ventricular systolic function**					
**LVEF, %**					
– Linear method	13.5 (7.8–23.8)	11.5 (6.0–20.9)	22.4 (15.0–34.1)	*<0*.*001*	*<0*.*001*
– Biplane	16.7 (10.0–28.5)	13.4 (9.6–26.3)	24.4 (15.8–35.5)	*<0*.*001*	*<0*.*001*
FS, %	6.0 (3.3–10.9)	4.8 (2.5–9.3)	9.5 (6.7–15.7)	*<0*.*001*	*<0*.*001*
LIMP	1.6 (1.1–2.3)	1.7 (1.2–2.4)	1.1 (0.8–1.6)	*<0*.*001*	*<0*.*001*
LVOT VTI, cm	4.8 (2.5–8.5)	4.4 (1.8–7.2)	7.7 (3.8–11.4)	*<0*.*001*	*<0*.*001*
s’—medial mitral annulus, m/s	0.030 (0.030–0.050)	0.030 (0.030–0.050)	0.040 (0.030–0.060)	*0*.*001*	*<0*.*001*
Lateral mitral annulus, m/s	0.040 (0.030–0.060)	0.040 (0.030–0.050)	0.050 (0.030–0.060)	*0*.*011*	*<0*.*001*
**Left ventricular diastolic function**					
E/A (if patient is in SR)	0.8 (0.5–1.1)	0.8 (0.6–1.1)	0.9 (0.6–1.1)	0.30	0.46
e’—medial, m/s^a^	0.030 (0.020–0.040)	0.030 (0.020–0.040)	0.030 (0.020–0.050)	0.002	*<0*.*001*
Lateral, m/s^a^	0.035 (0.030–0.050)	0.030 (0.020–0.040)	0.040 (0.030–0.050)	0.62	*<0*.*001*
E/e’- mean	11.7 (8.1–17.5)	10.8 (7.3–16.6)	10.5 (7.1–16.2)	1.00	0.75
**Hemodynamic parameters**					
SBP, mmHg	91 (76–104)	98 (80–108)	85 (71–101)	*0*.*013*	*<0*.*001*
DBP, mmHg	73 ± 17	71 ± 17	59 ± 15	*<0*.*001*	*<0*.*001*
MAP, mmHg	75 ± 18	77 ± 17	66 ± 19	*<0*.*001*	*<0*.*001*
HR, bpm	94 ± 20	92 ± 19	94 ± 19	0.55	0.06
Stroke volume, ml	15 (8–26)	12 (6–21)	23 (12–34)	*<0*.*001*	*<0*.*001*
Cardiac output, L/min	1.6 (0.8–2.3)	1.1 (0.5–2.3)	2.0 (1.2–3.1)	*<0*.*001*	*<0*.*001*
Cardiac index, L/min/m2	0.8 (0.5–1.3)	0.6 (0.3–1.3)	1.2 (0.7–1.7)	*<0*.*001*	*<0*.*001*
CPO, Watts	0.2 (0.1–0.4)	0.2 (0.1–0.4)	0.3 (0.2–0.5)	*<0*.*001*	*<0*.*001*
CPI, Watts/m2	0.1 (0.1–0.2)	0.1 (0–0.2)	0.2 (0.1–0.3)	*<0*.*001*	*<0*.*001*
**Strain values, %**					
Global longitudinal strain	−1.2 (−5.2–0)	0 (−3.8–0)	−2.8 (−7.6–0.1)	*<0*.*001*	*<0*.*001*
Longitudinal 3-chamber strain	−0.1 (−5.4–0)	0 (−5.3–0)	−3.4 (−7.8–0)	*0*.*006*	*<0*.*001*
Longitudinal 2-chamber strain	−0.1 (−4.7–0)	0 (−3.7–0)	−3.3 (−8.5–0)	*<0*.*001*	*<0*.*001*
Longitudinal 4-chamber strain	0 (−4.9–0)	0 (−4.6–0)	−3.3 (−7.0–0)	*0*.*016*	*0*.*001*

CPI, cardiac power index; CPO, cardiac power output; DBP, diastolic blood pressure; e’, early diastolic tissue velocity at mitral annulus; E/A, early to late diastolic transmitral flow velocity; E/e’, early diastolic transmitral flow velocity to e’; ECMO, extracorporeal membrane oxygenation; FS, fractional shortening; HR, heart rate; LIMP, left ventricular index of myocardial performance; LVEDV, left ventricular end-diastolic volume; LVEF, left ventricular ejection fraction; LVESV, left ventricular end-systolic volume; LVIDd, left ventricular internal diameter in diastole; LVIDs, left ventricular internal diameter in systole; LVOT, left ventricular outflow trace; MAP, mean arterial pressure; s’, peak systolic tissue velocity at mitral annulus; SBP, systolic blood pressure; VTI, velocity time integral.

^a^ Between group differences in medial and lateral e’ were tested by the Wilcoxon signed-rank tests and were statistically significantly different with *p* value < 0.05. The absolute differences in measurements were minimal.

### 3.6. Prediction of clinical outcomes

**Figure 3:**
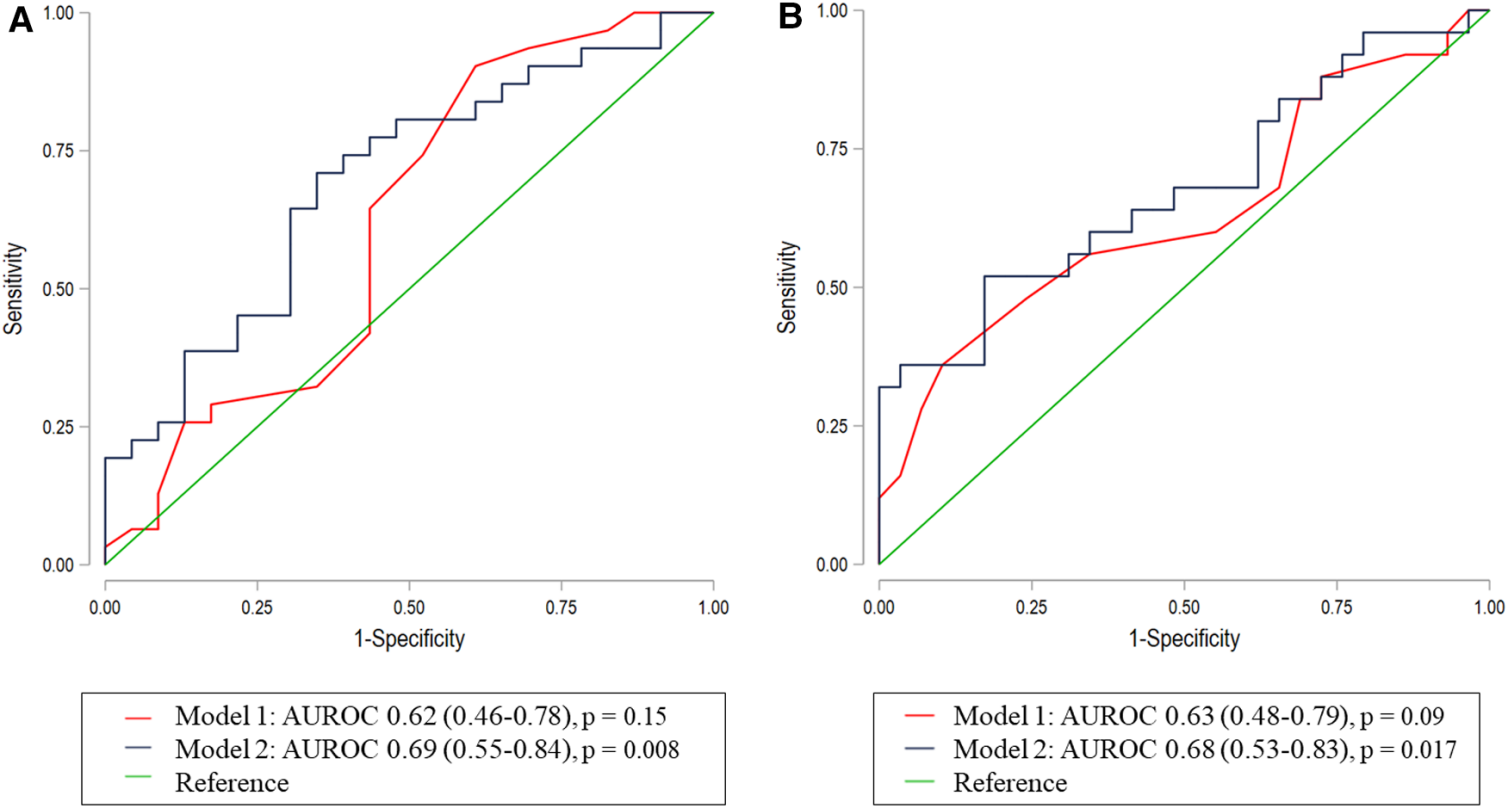
Receiver operating characteristic curves to predict hospital mortality and weaning Off ECMO. The receiver operative characteristic curves using Model 1 (SAVE score) (red), and Model 2 (SAVE score and echocardiographic parameters of left ventricular systolic function obtained at 100% ECMO target blood flow) (blue) to predict (**A**) hospital mortality and (**B**) successful weaning from V-A ECMO. Model 2 had the best discriminative ability with an area under the receiver operating characteristic curves of 0.69 (95% CI 0.55–0.84), *p* = 0.008 and 0.68 (95% CI 0.53–0.83), *p* = 0.017, respectively. *p-value compares to neutrality AUROC, Area under the receiver operating characteristic; V-A ECMO, veno-arterial extracorporeal membrane oxygenation.

The performance of 2 clinical models including data gathered early after ECMO implantation to predict eventual hospital mortality and successful weaning from V-A ECMO were examined. The addition of echocardiographic parameters of LV systolic function obtained at 100% TBF (Model 2) resulted in better discriminatory value compared with SAVE score only in predicting the hospital mortality (AUROC 0.69, 95% CI 0.55–0.84, *p* = 0.008 vs. 0.62, 95% CI 0.46–0.78, *p* = 0.15; respectively) and successful weaning from V-A ECMO (AUROC 0.68, 95% CI 0.53–0.83, *p* = 0.017 vs. 0.63, 95% CI 0.48–0.79, *p* = 0.09; respectively) (Figure 3). Hosmer-Lemeshow tests suggested that the models were well-calibrated (*p* > 0.05 for all).

### 3.7. Sensitivity analysis

7 (13.0%) patients who had IABP during ECMO were excluded from the subgroup analysis. All indices of LV systolic function including strain measurements were significantly better at 50% TBF were compared with 100% and 120% TBF, respectively (Table 3).

**Table 3:** Sensitivity analysis.

ECMO Target Blood Flow (*n* = 47)					
	100%	120%	50%	*p* value for 100% and 50%	*p* value for 120% and 50%
**Strain values, %**					
Global longitudinal strain	−1.2 (−5.2–0)	0 (−4.2–0)	−3.5 (−7.6–0)	*<0*.*001*	*<0*.*001*
**Left ventricular systolic function**					
**LVEF, %**					
– Biplane	16.8 (10.1–28.5)	13.1 (9.6–26.3)	24.0 (15.5–35.8)	*<0*.*001*	*<0*.*001*
FS, %	6.2 (3.3–10.9)	5.2 (2.5–9.3)	8.4 (6.4–15.7)	*<0*.*001*	*<0*.*001*
LIMP	1.6 (1.0–2.0)	1.5 (1.2–2.5)	1.1 (0.8–1.6)	*<0*.*001*	*<0*.*001*
LVOT VTI, cm	5.6 (2.7–8.7)	5.3 (2.0–7.5)	8.2 (3.9–11.7)	*<0*.*001*	*<0*.*001*
s’—medial mitral annulus, m/s	0.04 (0.03–0.05)	0.03 (0.03–0.05)	0.04 (0.03–0.06)	*<0*.*001*	*<0*.*001*
Lateral mitral annulus, m/s	0.04 (0.03–0.06)	0.04 (0.03–0.06)	0.05 (0.03–0.07)	*0*.*007*	*<0*.*001*

ECMO, extracorporeal membrane oxygenation; FS, fractional shortening; LIMP, left ventricular index of myocardial performance; LVEF, left ventricular ejection fraction; LVOT, left ventricular outflow trace; s’, peak systolic tissue velocity at mitral annulus; VTI, velocity time integral.

## 4. Discussion

In a prospective cohort of patients on V-A ECMO, the use of two-dimensional strain provided robust echocardiographic evidence of the afterload effect of peripheral V-A ECMO support during the acute phase of myocardial injury. Across different echocardiographic parameters, decreasing ECMO blood flow was associated with significantly measures increased LV systolic function. These physiological data are fundamental to understanding the heart-ECMO interaction, interpreting echocardiographic measures of LV function, guiding titration of ECMO blood flow, and optimizing cardiac recovery.

The prevalent beliefs about hemodynamics during peripheral V-A ECMO include a decrease in the SV and cardiac output due to reduction in native cardiopulmonary circulation and an increase in the LV afterload (4, 11). Depending on the competing effects on decreasing preload and increasing afterload, the LV chamber size, systolic function, and LV stroke work may be variable. In a pilot study including 22 patients by Aissaoui et al., it was shown that the LVEF, VTI, and LV strain were all load dependent (12). The authors found that tissue Doppler velocities were more useful than LV strain for characterizing the LV function, but broad generalization of the study findings are limited by the cohort having included both central and peripheral V-A ECMO configurations. Our cohort included a homogenous group of patients supported on peripheral V-A ECMO, who, by nature of the retrograde return of blood flow in the descending aorta, are particularly sensitive to load variations induced by changes in ECMO flow settings. All measures of LV systolic function were better with the ECMO blood flow set at 50% of target, including increase in LV GLS, increase in LVEF, increase in LVOT VTI, and increase in s' at the mitral annulus. Together with the lower MAP, higher stroke volume, and higher cardiac index observed during reduced ECMO blood flow, our data highlights that management of patients on ECMO requires a thorough assessment of hemodynamic and echocardiographic indices.

The optimization of ECMO blood flow for the heterogeneous conditions necessitating V-A ECMO is a complex issue, with sparse evidence for specific targets in published literature. Considering that initial ECMO set up is largely titrated to a target flow, this “ECMO dose” should be better delineated. While a general target ECMO flow of 50–80 ml/kg/min have been proposed (7, 8), others have emphasized targeting mean arterial pressure or venous oxygen saturation to ensure adequate oxygen delivery (13, 14). There are increasing data to suggest improved outcomes after concurrent LV unloading during V-A ECMO (15), with marked reduction of the pulmonary venous pressure, potentially reducing the degree of pulmonary edema and limiting myocardial infarct size (3). However, few studies have provided direct echocardiographic evidence of depressed LV systolic function that were correlated with higher ECMO flows in a dose-dependent manner. Whether “permissively low” ECMO blood flows should be targeted after ECMO implantation in return for better LV systolic function deserves careful consideration, taking into account other physiological effects of ECMO at lower flows such as increased hemolysis in the centrifugal pump (16).

We highlighted that quantitative echocardiography in the management of patients requiring V-A ECMO extends beyond assessment of myocardial function at stably maintained ECMO flows (17). Recent data have emerged that dynamic indices such as improvement in tissue Doppler velocities predict ECMO weanability (18). The interplay between LV performance metrics and different levels of ECMO blood flow needs to be mapped out for the individual patient, and repeated profiling should be attempted throughout the course of ECMO support. While most conventional measures of LV systolic function could be used in patients with V-A ECMO, myocardial strain has emerged as a less load-dependent measurement that is more reproducible than LVEF, even when performed by less-experienced operators (19). Moreover, estimation of LVEF with the Teichholz formula is no longer recommended and may even be misleading in regional LV hypokinesia. These properties make strain imaging an attractive tool for repeated assessment at the point-of-care and should be considered by all ECMO providers in the ICU (20). Together with a thoughtful use of the invasive pulmonary arterial catheter, continuous flow data that guides ECMO flow titration and decisions to vent or wean can be made.

The SAVE score is one of the most widely accepted risk prediction models to estimate hospital survival for patients on V-A ECMO (10). However, it performed modestly in our cohort, possibly due to the significant proportion of patients who had received ECMO during ECPR, who were not considered in the original development of the SAVE score. ECPR-specific scores have since been published (21), but their widespread adoption is limited by the lack of external validation. We showed that by incorporating echocardiographic derivatives of LV function, the performance of the SAVE score could be improved, with a benefit to be broadly applicable to all V-A ECMO patients, regardless of ECPR status. It is also possible that repeated two-dimensional and strain measurements obtained later in the course of ECMO support may be more correlated with eventual outcomes. Validation of updated risk scores should be a priority for future collaborative international multicenter studies.

One of the limitations of this study is its single-centered design, with possible systemic biases in patient selection and management. However, the inclusion criteria were not restrictive, and this is one of the larger V-A ECMO cohorts with detailed echocardiographic documentation of the heart-ECMO interaction at various flow rates. Second, patients who were hemodynamically unstable during the first 48 h and patients who had LV venting had to be excluded by nature of the study design, potentially limiting the application of the study findings to these patients. Third, other aspects of patient management, including the use of inotropes and titration of positive end-expiratory pressure were not protocolized, possibly introducing variable effects on preload and afterload.

## 5. Conclusions

We provided echocardiographic data to demonstrate the inverse effects of ECMO blood flow on LV systolic function in a dose-dependent manner. These physiological data highlight the significance of determining goals of ECMO flow after initiation. Two-dimensional strain should be considered a promising tool for repeatable assessment of LV function for patients on V-A ECMO.

## Data Availability

The raw data supporting the conclusions of this article will be made available by the authors, without undue reservation.
