# Supplementary material for: Effects of varying blood flow rate during peripheral veno-arterial extracorporeal membrane oxygen (V-A ECMO) on left ventricular function measured by two-dimensional strain
Source: Front Cardiovasc Med. 2023 Apr 12;10:1147783. doi: 10.3389/fcvm.2023.1147783 (PMC10130508; doi:10.3389/fcvm.2023.1147783)
Supplement: Supplementary file 1 [file Datasheet1.docx]

Supplementary Material

**Effects of varying blood flow rate during peripheral veno-arterial extracorporeal membrane oxygen (V-A ECMO) on left ventricular function measured by two-dimensional strain**

**Pauline Yeung Ng**, **Tammy Sin Kwan Ma**, **April Ip**, **Shu Fang**, **Andy Chak Cheung Li**, **Alfred Sai Kuen Wong**, **Chun Wai Ngai**, **Wai Ming Chan**, **Wai Ching Sin***

* **Correspondence:** Wai Ching Sin: [drwcsin@hku.hk](mailto:drwcsin@hku.hk)

**Table S1. Two-dimensional echocardiographic measurements and normal values**

| **Measurement** | **Calculations** | **Normal value** |
| --- | --- | --- |
| **Left heart volume** | | |
| LVEDV, mL | Biplane method of disks summation | 76±15 in female  106±22 in male |
|  |  |  |
| LVESV, mL | Biplane method of disks summation | 28±7 in female  41±10 in male |
|  | | |
| **Left ventricular function** | | |
| LVEF, % | Linear method  Biplane method | 64±5 in female  62±5 in male |
|  |  |  |
| FS, % | 2D linear measurement | 27-45 in female  25-43 in male |
| LIMP | Global estimate of both systolic and diastolic function | <0.40 |
| S’, cm/s | Peak systolic tissue velocity at the mitral annulus | >10 |
|  | | |
| **Hemodynamic data** | | |
| SV, mL | LVOT CSA x LVOT VTI | 60-100 |
| CI, L/min/m^2^ | $\frac{Cardiac output}{Body Surface Area}$ | 2.4-4.0 |
| CPI, Watts/ m^2^ | $\frac{Cardiac index x Mean Arterial Pressure}{451}$ | >0.6 |
|  | | |
| **Strain** | | |
| GLS, % | Average of longitudinal strain in 3-,4- and 2- chamber views | 17.3-21.5 |

**Abbreviations:** CI: cardiac index; CPI: cardiac power index; CSA: cross sectional area; FS: fractional shortening; GLS: global longitudinal strain; LIMP: left index of myocardial performance; LVEDV: left ventricular end diastolic volume; LVEF: left ventricular ejection fraction; LVESV: left ventricular end systolic volume; LVOT: left ventricular outflow tract; S’: peak systolic annulus velocity; SV: stroke volume; VTI: velocity time integral.

| **Table S2. Clinical Parameters of the study population** | |  |
| --- | --- | --- |
| **Hemodynamic parameters**  **(worst values on day 1 of ECMO)** | **N=54** | |
| Mean arterial pressure, mmHg | 55 (45-76) | |
| Heart rate, beats per minute | 115 (101-126) | |
|  |  | |
| **Vasopressor/ inotrope use**  **(maximum dose on day 1 of ECMO)** |  | |
| -         Noradrenaline, mcg/min/kg | 0.3 (0.1-0.4) | |
| -         Adrenaline, mcg/min/kg | 0.2 (0.1-0.2) | |
| -         Dobutamine, mcg/min/kg | 6.1 (2.6-10.2) | |
| -         Dopamine, mcg/min/kg | 15.4 (11.0-21.0) | |
|  |  | |
| **Biochemistry (worst values on day 1 of ECMO)** |  | |
| **Renal** |  | |
| -         Creatinine, umol/L | 204.5 (136.0-294.0) | |
| -         Arterial blood pH | 7.1 ± 0.2 | |
| -         Bicarbonate, mmol/L | 14.2 ± 5.6 | |
| -         Base excess, mmol/L | -15.8 ± 7.4 | |
| **Liver** |  | |
| -         Bilirubin, umol/L | 19.8 (11.0-30.0) | |
| -         Alanine transaminase U/L | 191.0 (90.0-547.0) | |
| -         International normalized ratio | 1.6 (1.3-2.5) | |
| **Hematological** |  | |
| -         Hemoglobin, g/dL | 10.5 ± 2.0 | |
| -         White cell count, x10^9^/L | 18.1 (15.8-21.9) | |
| -         Platelet, x10^9^/L | 133.6 ± 72.6 | |
| **Respiratory** |  | |
| -         PaO2, kPa | 10.0 (7.6-14.1) | |
| -         PaCO2, kPa | 7.7 (6.4-11.0) | |
| **Cardiovascular** |  | |
| -         Creatine Kinase, U/L | 806.0 (224.0-3,124.0) | |
| -         Troponin T, ng/L | 7,680 (1,470-23,634) | |
| -         Lactate, mmol/L | 11.6 ± 5.9 | |
|  |  | |

All data are presented as frequency with percentages or mean ± standard deviation, or median with interquartile range (IQR) unless specified.

**Abbreviations:** ECMO: extracorporeal membrane oxygenation; PaO2: partial pressure of oxygen; PaCO2: partial pressure of carbon dioxide.

**Table S3. Clinical Outcomes Stratified by the SAVE Score**

The Survival After Veno-arterial ECMO (SAVE) score is a tool to predict hospital survival for patients receiving V-A ECMO. We examined the outcomes of our patients stratified by the SAVE score.

| **SAVE groups** | **SAVE score** | **Number (%)** | **Hospital Mortality  (% of group)** |
| --- | --- | --- | --- |
| I | >5 | 2 (3.7%) | 0 |
| II | 1 to 5 | 1 (1.9%) | 0 |
| III | -4 to 0 | 21 (38.9%) | 11 (52.4%) |
| IV | -9 to -5 | 19 (35.2%) | 12 (63.2%) |
| V | <= -10 | 11 (20.4%) | 8 (72.7%) |

We examined whether LV strain would predict the different SAVE groups in a multinomial regression model – LV strain was not significantly associated with SAVE groups (P=0.85, 0.23, 0.60, 0.43; respectively).

**Table S4. Comparison of Echo Findings at Different Levels of ECMO Target Blood Flow by Hospital Mortality**

|  | **Survivors (n=23)** | | |  | **Dead (n=31)** | | |  | **p-value of survivors vs. dead** | | |
| --- | --- | --- | --- | --- | --- | --- | --- | --- | --- | --- | --- |
| **Variables** | **100% ECMO flow** | **120% ECMO flow** | **50%**  **ECMO flow** |  | **100% ECMO flow** | **120% ECMO flow** | **50%**  **ECMO flow** |  | **100%**  **ECMO Flow** | **120%**  **ECMO Flow** | **50%**  **ECMO Flow** |
| **Left ventricle size** | | | | | | | | | | | |
| **LVIDd, cm** | 4.7  (4.0-5.2) | 4.6  (3.5-5.2) | 4.3  (3.5-5.1) |  | 3.9  (3.3-4.7) | 3.9  (3.2-4.7) | 3.8  (3.1-4.5) |  | *0.004* | 0.15 | 0.08 |
| **LVIDs, cm** | 4.4  (3.4-5.0) | 4.1  (3.0-5.0) | 3.9  (3.0-4.9) |  | 3.5  (3.0-4.6) | 3.6  (3.1-4.5) | 3.4  (2.5-4.2) |  | 0.07 | 0.23 | 0.15 |
| **LVEDV, mL** | 77.0  (64.2-133.5) | 94.7  (45.3-138.8) | 82.4  (42.2-148.7) |  | 68.3  (33.8-102.6) | 75.0  (42.1-105.6) | 74.1  (29.6-104.2) |  | 0.14 | 0.19 | 0.12 |
| **LVESV, mL** | 60.1  (46.8-131.6) | 70.7  (40.0-112.2) | 51.8  (30.9-120.9) |  | 54.0  (27.2-88.9) | 55.4  (32.0-87.4) | 51.4  (22.3-85.8) |  | 0.18 | 0.26 | 0.18 |
|  |  |  |  |  |  |  |  |  |  |  |  |
| **Left ventricular systolic function** | | | | | | | | | | | |
| **LVEF, %** | | | | | | | | | | | |
| **-   Linear method** | 13.8  (7.1-27.2) | 12.5  (5.3-23.9) | 22.8  (14.4-35.9) |  | 13.4  (8.5-23.1) | 10.3  (6.1-20.9) | 22.0  (15.0-31.9) |  | 0.89 | 0.99 | 0.91 |
| **-   Biplane** | 16.8  (10.0-31.9) | 12.4  (9.6-27.4) | 29.0  (17.6-34.8) |  | 16.1  (9.5-28.5) | 13.7  (8.0-26.3) | 24.0  (15.5-35.8) |  | 0.81 | 0.95 | 0.80 |
| **FS, %** | 6.2  (3.2-12.6) | 5.3  (2.4-10.4) | 10.5  (6.4-17.3) |  | 5.8  (3.7-9.9) | 4.5  (2.7-9.3) | 8.4  (6.7-14.8) |  | 0.90 | 0.90 | 0.98 |
| **LIMP** | 1.7  (1.0-2.0) | 1.7  (1.2-2.4) | 1.0  (0.8-1.3) |  | 1.6  (1.1-2.3) | 1.7  (1.2-2.4) | 1.2  (0.9-2.0) |  | 0.40 | 0.81 | 0.15 |
| **LVOT VTI, cm** | 5.7  (2.9-9.6) | 5.5  (1.8-8.8) | 8.7  (5.5-13.4) |  | 4.6  (2.2-7.9) | 3.5  (1.0-6.3) | 6.1  (3.5-11.4) |  | 0.17 | 0.20 | 0.16 |
| **s’ – medial mitral annulus, m/s** | 0.04  (0.03-0.05) | 0.03  (0.03-0.05) | 0.04  (0.03-0.05) |  | 0.03  (0.02-0.06) | 0.03  (0.03-0.05) | 0.04  (0.03-0.06) |  | 0.67 | 0.67 | 0.96 |
| **s’ – lateral mitral annulus, m/s** | 0.05  (0.03-0.06) | 0.04  (0.03-0.05) | 0.06  (0.03-0.07) |  | 0.04  (0.03-0.06) | 0.04  (0.03-0.06) | 0.04  (0.03-0.06) |  | 0.64 | 0.88 | 0.52 |
|  |  |  |  |  |  |  |  |  |  |  |  |
| **Left ventricular diastolic function** | | | | | | | | | | | |
| **E/A (if patient is in SR)** | 0.8  (0.6-1.2) | 0.8  (0.6-1.1) | 0.9  (0.6-1.2) |  | 0.9  (0.5-1.1) | 0.8  (0.6-1.1) | 0.8  (0.7-0.9) |  | 0.94 | 0.98 | 0.52 |
| **e’ – medial, m/s** | 0.02  (0.02-0.04) | 0.03  (0.02-0.04) | 0.03  (0.02-0.05) |  | 0.03  (0.02-0.04) | 0.03  (0.02-0.03) | 0.03  (0.02-0.05) |  | 0.74 | 0.69 | 0.93 |
| **e’ –lateral, m/s** | 0.04  (0.03-0.05) | 0.03  (0.02-0.05) | 0.04  (0.03-0.05) |  | 0.03  (0.03-0.05) | 0.03  (0.02-0.04) | 0.03  (0.02-0.05) |  | 0.13 | 0.46 | 0.50 |
| **E/e’- mean** | 12.8  (8.3-17.5) | 13.3  (8.7-16.6) | 12.6  (10.2-20.4) |  | 11.5  (7.7-17.9) | 8.5  (7.3-17.5) | 8.3  (6.4-13.4) |  | 0.75 | 0.24 | *0.013* |
|  |  |  |  |  |  |  |  |  |  |  |  |
| **Hemodynamic parameters** | | | | | | | | | | | |
| **SBP, mmHg** | 98  (77-105) | 98  (79-105) | 85  (73-98) |  | 89  (75-100) | 99  (83-115) | 85  (67-107) |  | 0.43 | 0.61 | 0.79 |
| **DBP, mmHg** | 75±18 | 69±14 | 60±13 |  | 71±16 | 72±18 | 59±17 |  | 0.48 | 0.45 | 0.85 |
| **MAP, mmHg** | 77±15 | 77±14 | 69±14 |  | 73±19 | 78±20 | 64±21 |  | 0.41 | 0.76 | 0.37 |
| **HR, bpm** | 88±19 | 85±20 | 91±21 |  | 98±19 | 97±18 | 97±18 |  | 0.068 | *0.024* | 0.32 |
| **Stroke volume, mL** | 18  (10-29) | 16  (7-29) | 27  (17-35) |  | 13  (6-25) | 10  (3-19) | 18  (11-34) |  | 0.13 | 0.13 | 0.12 |
| **Cardiac output, L/min** | 1.6  (1.0-2.5) | 1.1  (0.6-2.4) | 2.5  (1.3-3.2) |  | 1.3  (0.7-2.3) | 1.1  (0.3-1.8) | 1.8  (1.1-3.1) |  | 0.36 | 0.39 | 0.30 |
| **Cardiac index, L/min/m2** | 0.8  (0.5-1.4) | 0.5  (0.3-1.4) | 1.3  (0.7-1.7) |  | 0.8  (0.4-1.3) | 0.6  (0.2-1.0) | 1.0  (0.7-1.8) |  | 0.42 | 0.47 | 0.40 |
| **CPO, Watts** | 0.3  (0.2-0.4) | 0.2  (0.1-0.4) | 0.4  (0.2-0.5) |  | 0.2  (0.1-0.4) | 0.2  (0.1-0.3) | 0.3  (0.1-0.5) |  | 0.25 | 0.43 | 0.16 |
| **CPI, Watts/m2** | 0.1  (0.1-0.3) | 0.1  (0-0.2) | 0.2  (0.1-0.3) |  | 0.1  (0-0.2) | 0.1  (0-0.2) | 0.2  (0.1-0.3) |  | 0.37 | 0.53 | 0.27 |
|  |  |  |  |  |  |  |  |  |  |  |  |
| **Strain values, %** | | | | | | | | | | | |
| **Global longitudinal strain** | -2.1  (-5.4-0) | 0  (-4.2-0) | -3.5  (-8.2--0) |  | -1.1  (-5.1-0) | 0  (-3.3-0) | -1.9  (-7.4--0.1) |  | 0.53 | 0.52 | 0.87 |
| **Longitudinal 3-chamber strain** | -3.5  (-6.1-0) | 0  (-5.0-0) | -4.0  (-9.3-0) |  | 0  (-5.4-0) | 0  (-5.5-0) | -3.2  (-7.0-0) |  | 0.47 | 0.92 | 0.78 |
| **Longitudinal 2-chamber strain** | -0.1  (-6.0-0) | 0  (-3.8-0) | -4.3  (-8.7-0) |  | -0.1  (-4.3-0) | 0  (-3.1-0) | -3.2  (-6.5-0) |  | 0.72 | 0.69 | 0.63 |
| **Longitudinal 4-chamber strain** | 0  (-4.7-0) | 0  (-5.0-0) | -3.3  (-6.4-0) |  | 0  (-5.0-0) | 0  (-4.6-0) | -3.2  (-7.8-0) |  | 0.85 | 0.43 | 0.90 |

**Abbreviations:** CPI: cardiac power index; CPO: cardiac power output; DBP: diastolic blood pressure; e’: early diastolic tissue velocity at mitral annulus; E/A: early to late diastolic transmitral flow velocity; E/e’: early diastolic transmitral flow velocity to e’; ECMO: extracorporeal membrane oxygenation; FS: fractional shortening; HR: heart rate; LIMP: left ventricular index of myocardial performance; LVEDV: left ventricular end-diastolic volume; LVEF: left ventricular ejection fraction; LVESV: left ventricular end-systolic volume; LVIDd: left ventricular internal diameter in diastole; LVIDs: left ventricular internal diameter in systole; LVOT: left ventricular outflow trace; MAP: mean arterial pressure; s’: peak systolic tissue velocity at mitral annulus; SBP: systolic blood pressure; VTI: velocity time integral.
